# An Integrated View of the Effects of Wine Polyphenols and Their Relevant Metabolites on Gut and Host Health

**DOI:** 10.3390/molecules22010099

**Published:** 2017-01-06

**Authors:** Carolina Cueva, Irene Gil-Sánchez, Begoña Ayuda-Durán, Susana González-Manzano, Ana María González-Paramás, Celestino Santos-Buelga, Begoña Bartolomé, M. Victoria Moreno-Arribas

**Affiliations:** 1 Instituto de Investigación en Ciencias de la Alimentación (CIAL), CSIC-UAM. Nicolás Cabrera, 9. Campus de Cantoblanco, 28049 Madrid, Spain; carolina.cueva@csic.es (C.C.); irene.gil.sanchez@cial.uam-csic.es (I.G.-S.); b.bartolome@csic.es (B.B.); 2 Grupo de Investigación en Polifenoles, Universidad de Salamanca, Campus Miguel de Unamuno, 37007 Salamanca, Spain; Bego_Ayuda@usal.es (B.A.-D.); susanagm@usal.es (S.G.-M.); paramas@usal.es (A.M.G.-P.); csb@usal.es (C.S.-B.)

**Keywords:** wine polyphenols, flavonoids, gut microbiota, microbial phenolic metabolites, inter-individual variability

## Abstract

Over the last few decades, polyphenols, and flavonoids in particular, have attracted the interest of researchers, as they have been associated with the health-promoting effects derived from diets rich in vegetables and fruits, including moderate wine consumption. Recent scientific evidence suggests that wine polyphenols exert their effects through interactions with the gut microbiota, as they seem to modulate microbiota and, at the same time, are metabolized by intestinal bacteria into specific bioavailable metabolites. Microbial metabolites are better absorbed than their precursors and may be responsible for positive health activities in the digestive system (local effects) and, after being absorbed, in tissues and organs (systemic effects). Differences in gut microbiota composition and functionality among individuals can affect polyphenol activity and, therefore, their health effects. The aim of this review is to integrate the understanding of the metabolism and mechanisms of action of wine polyphenols at both local and systemic levels, underlining their impact on the gut microbiome and the inter-individual variability associated with polyphenols’ metabolism and further physiological effects. The advent of promising dietary approaches linked to wine polyphenols beyond the gut microbiota community and metabolism are also discussed.

## 1. Introduction

Among polyphenol-rich food, wine—and in particular red wine—has attracted great interest from the scientific community, especially since different epidemiological and randomized clinical intervention trials, such as the International MONICA (MONItoring of trends and determinants in CArdiovascular disease) study [1] and more recently the PREDIMED (PREvención con DIeta MEDiterrénea) Project [1], revealed that moderate wine consumption as part of a healthy diet and a lifestyle is associated with protective effects against relevant chronic diseases. Indeed, despite wine ethanol content, it is well documented in peer-reviewed epidemiological surveys and in vitro animal and, to a lesser extent, human studies that moderate consumption of wine is associated with a reduced risk of developing cardiovascular diseases (CVD), diabetes, and neurodegenerative disorders, among others [1,2]. These effects have mostly been attributed to the polyphenol composition of wine. The amount of polyphenols in wine, although varying greatly, is estimated to be around 150–400 mg/L in white wines and 900–1400 mg/L in young red wines [3]. Red wine provides a unique combination of phenolic structures, including flavonoids as the main compounds, namely flavan-3-ol monomers (catechin and epicatechin), oligomers and polymers (proanthocyanidins or condensed tannins), anthocyanins (malvidin-3-*O*-glucoside, mainly) and flavonols (quercetin, myricetin and kaempferol and their glycosides), but also non-flavonoid compounds, such as hydroxybenzoic and hydroxycinnamic acids, phenolic alcohols, stilbenes, and ellagitannins.

**Figure 1:**
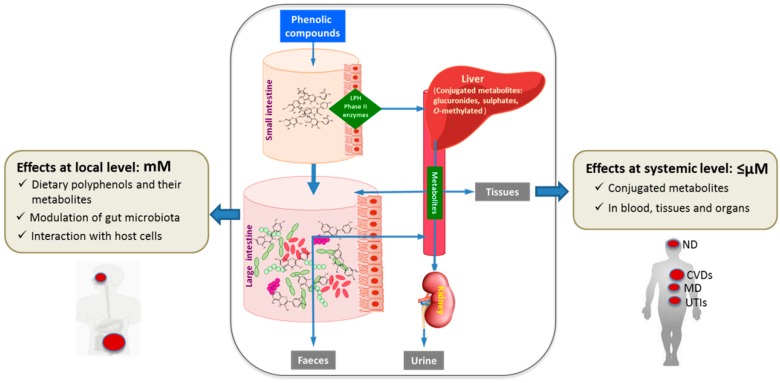
Integrated overview of the metabolism and health effects of dietary polyphenols. Abbreviations. ND: neurological disease; CVDs: cardiovascular diseases; MD: metabolic disorders; UTIs: urinary tract infections.

The role of dietary polyphenols in human health depends largely on their bioavailability, absorption, and metabolism [4]. Once ingested, it has been estimated that only 5%–10% of the total polyphenol intake could be absorbed in the small intestine, whereas 90%–95% would reach the colon [5] where the polyphenols are transformed by the resident microbiota into bioavailable metabolites that might be even more bioactive than their precursors (Figure 1) [6,7]. An individual variability in metabolite production has been reported, depending on the specific chemical structure of the polyphenol and differences/variations in gut microbiota [8,9].

Dietary polyphenols and/or their metabolites may exert beneficial health effects at a local level, directly during their passage through the oral cavity and gastrointestinal tract, and at a systemic level, after being absorbed. The actual concentrations of phenolic metabolites that can be found in the human intestinal lumen (local effect) under realistic polyphenol consumption may reach up to the millimolar range. Modulation of intestinal microbiota, anti-inflammatory effects, and modulation of immune function could be the most remarkable beneficial local health effects exerted by polyphenols and their microbial metabolites. A fraction of them could also be absorbed and found in plasma in the form of phenolic conjugates in levels that may range from nanomolar to low micromolar range [10]. At the systemic level, potential cardio- and neuro-protective effects of polyphenols have been described, as well as a beneficial role in the prevention of some types of cancer, diabetes, inflammatory diseases, and metabolic disorders [11,12,13].

It is accepted that a two-way interaction between polyphenols and gut microbiota exists, so that not only is the gut microbiome involved in the metabolism of wine polyphenols, but also the phenolic compounds and their microbial metabolites may impact on gut microbiota and induce prebiotic-like effects on bacteria. The human gastrointestinal tract is home to a unique ecosystem of microorganisms, where Firmicutes (including *Clostridium*, *Enterococcus*, *Lactobacillus*, *Ruminococcus,* and *Faecalibacterium* genera) and Bacteroidetes (including *Bacteroides* and *Prevotella* genera) are the dominant bacterial phyla [14]. The adult-like intestinal microbiota is regarded as relatively stable throughout adulthood, until old age [15]. However, several studies have shown that extrinsic factors, such as diet and antibiotics, induce transient fluctuations in the gut microbiota. Due to the crucial role of the gut microbiota in human health, imbalances in its composition and/or function (dysbiosis) are recognized as possible causes of intestinal, metabolic, and autoimmune diseases [16]. Several strategies, including the intake of some types of polyphenols, have been proposed to modulate the composition and metabolic activity of the human gut microbiota [17]. Indeed, the study of the effects of (wine) polyphenols on the gut microbiota needs a multidisciplinary approach that takes into account the complexity of the human microbiome and its metabolic function, variability among different population groups, and coexistence with other bioactive dietary components.

The aim of the present review is to compile the updated information on the metabolism and mechanisms of action of wine polyphenols in the human body at both local and systemic levels, with particular emphasis on their impact on gut microbiota composition and functionality, and the inter-individual variability associated with their metabolism and further physiological effects. Finally, potential emerging dietary strategies related to wine polyphenols are discussed.

## 2. Metabolism of Wine Polyphenols

The first step of the digestion of polyphenols takes place in the oral cavity. During mastication, food is broken up into small portions and also mixed with both saliva and oral microbiota enzymes, such as glycosidases, which may lead to the hydrolysis of some flavonoid-*O*-glycosides [18]. In the transit through the digestive tract into the stomach, the size of food particles is reduced, enhancing the release of phenolic compounds from the food matrix [19]. In the small intestine, flavonoid glycosides may be hydrolyzed by lactase phloridzin hydrolase (LPH) in the epithelial cells of the brush-border, and the resulting aglycones enter the enterocyte by passive diffusion [20]. It has been suggested that some particular flavonoids might also be transported into epithelial cells by the active sodium-dependent glucose transporter SGLT1 and be hydrolyzed in the cell by a cytosolic β-glucosidase (CBG) [21]. During transfer across the enterocyte, polyphenols undergo phase II conjugation reactions (glucuronidation, sulphation, and *O*-methylation), and further, extended conversion takes place in the liver, where enterohepatic transport in the bile may also occur and some conjugated metabolites are recycled back to the small intestine [22]. Conjugation reactions might also involve glutathione (GSH), which is present in significant levels in most tissues. This reaction may occur either spontaneously or be catalyzed by the phase II enzymes glutathione *S*-transferases (GSTs) [23].

Indeed, the bioavailability of polyphenols is very low. As for flavan-3-ols and anthocyanins, that constitute the majority of the phenolic compounds in red wine, the concentrations that could be expected to occur in plasma under a realistic dietary intake are in the nanomolar to low micromolar range [24]. Still lower, poorer bioavailability exists for resveratrol, for which it has been shown that consumption of amounts as high as 1.0 g would afford maximal plasma concentrations of ~0.6 μM in humans [25]. This low bioavailability linked to the very small concentrations of resveratrol usually present in wine (average contents of 1.9 ± 1.7 mg trans-resveratrol/L, ranging from non-detectable levels to 14.3 mg/L) [26] makes it unlikely that resveratrol is a relevant contributor to the putative healthy effects associated to wine consumption.

Compounds not absorbed in the small intestine, together with those recycled by enterohepatic circulation, reach the large intestine where they undergo extensive metabolism by the gut microbiota (Table 1). Microbial catabolism pathways of the different flavonoid classes (anthocyanins, flavonols, flavan-3-ols, etc.) are known to share similar intermediate and end products. Microbial-derived metabolites may be absorbed in the large intestine and then further metabolized in the liver by phase II enzymes into conjugated metabolites (glucuronides and sulphates), which can be distributed to the tissues, come back to the intestine, or be excreted in urine [27]. In the following paragraphs, the microbial metabolism of the main classes of phenolic compounds present in wine are described.

Flavan-3-ols: The catabolism of catechins and oligomeric proanthocyanidins involves the C-ring opening, followed by different reactions like lactonization, decarboxylation, dehydroxylation, and oxidation [28]. In the case of galloyled monomeric flavan-3-ols (e.g., epicatechin-3-*O*-gallate), the microbial catabolism usually starts with the rapid cleavage of the gallic acid ester moiety by microbial esterases, releasing gallic acid that is further decarboxylated into pyrogallol [29,30]. When opened, the C-ring gives rise to 1-(3′,4′-dihydroxyphenyl)-3-(2″,4″,6″-trihydroxyphenyl)-propan-2-ol, which is later converted into 5-(3′,4′-dihydroxyphenyl)-γ-valerolactone in the case of (epi)catechin, or 5-(3′,4′,5′-trihydroxyphenyl)-γ-valerolactone in that of (epi)gallocatechin [31]. The valerolactone ring may later break, leading to the formation of 5-(3′,4′-dihydroxyphenyl)valeric acid and/or 4-hydroxy-5-(3′,4′-dihydroxyphenyl)valeric acid. Phenyl-γ-valerolactones and phenylvaleric acids have been described as exclusive microbial metabolites of flavan-3-ols. Subsequent biotransformations of these valeric acids may give rise to hydroxyphenylpropionic and hydroxybenzoic acids by successive loss of carbon atoms from the side chain through β-oxidation [30]. Furthermore, other minor catabolites, such as hippuric acid, *p*-coumaric acid, vanillic acid, homovanillic acid, homovanillyl alcohol, and 3-O-methylgallic acid have been associated with the in vivo colonic metabolism of flavan-3-ols [32] (Table 1).

Flavonols: Quercetin 3-*O*-glucoside is the most representative flavonol in wines. During the first steps of the colonic catabolism of the aglycone, dihydroquercetin (taxifolin) is formed, which is further metabolized to 3-(3,4-dihydroxyphenyl)propionic acid (Table 1). These metabolites then enter the catabolic route of phenyl acids and benzoic acids to generate protocatechuic acid [33] and 2-(3,4-dihydroxy)-phenylacetic acid [34] as the majority metabolites (Table 1).

Anthocyanins: After microbial deglycosylation, ring fission of the aglycone takes place, releasing two parts: one from the A-ring and the other from the B-ring [35]. From the fragment corresponding to the B-ring, the production of different phenolic acids has been described [36,37,38]. According to those studies, the generation of benzoic acids, such as 3,4-dihydroxy-, 4-hydroxy-, 3,4-dimethoxy- or 3-methoxyl-4-hydroxyl benzoic acids, prevails over other types of phenolic acids (Table 1). The fragment corresponding to the A-ring of anthocyanins generates 2,4,6-trihydroxybenzaldehyde [39], which could further be converted to phloroglucinol [40] (Table 1).

On the other hand, both flavan-3-ols and anthocyanins can undergo different chemical reactions during winemaking leading to new products such as pyranoanthocyanins [41]. Although there are still not many studies about them, it has been proven absorption of flavanol-anthocyanin adducts in intestinal Caco-2 cells [42].

Stilbenes: Resveratrol is the most common stilbene present in grapes and wine. A study conducted by Bode et al. [43] showed that the main gut microbial metabolites of resveratrol are dihydroresveratrol, 3,4′-dihydroxy-trans-stilbene, and 3,4′-dihydroxybibenzyl (lunularin).

Ellagitannins: These compounds are the main class of hydrolysable tannins present in wines. They undergo degradation by gut microbiota releasing ellagic acid, which is further metabolized to give rise to urolithin A and its monohydroxylated analogue known as urolithin B [40].

The main phenolic metabolites found in urine and plasma after intake of wine polyphenols are glucuronides, sulphates, and methylated derivatives of flavan-3-ols such as anthocyanins and flavonols as well as metabolites derived from their microbial catabolism, mainly phenolic acids that can also be found in conjugated forms (for a review, see [44]). Also, recent studies have reported that the fecal phenolic profile in feces after wine consumption is mainly composed of microbial-derived phenolic acids and other related metabolites derived from the main classes of wine polyphenols [45,46].

It is important to remark that there is a great inter-individual variation in the profile and/or content of phenolic metabolites in physiological fluids (urine, plasma, feces) after a controlled intake of phenolic-rich foods [47,48]. Actually, in the particular case of ellagitannins, individual “metabotype” stratification has been proposed according to their ability to produce urolithins, the specific polyphenol-derived gut microbiota metabolites [49]. In the case of wine polyphenols, a tentative distribution of the individuals into low, medium, and high metabolizers by their capacity to metabolize wine polyphenols has been established [46], which has been attributed to variations in the activity of the intestinal microbiota and other metabolic pathways [50].

## 3. Effects and Mechanisms of the Action of Wine Polyphenols at the Systemic Level

The potential health benefits of flavonoids, and polyphenols in general, have been classically attributed to their scavenging/antioxidant activity, which might help counteract oxidative damage in cells and tissues. Actually, many polyphenols have demonstrated in vitro to be effective scavengers of most types of oxidizing species, such as superoxide anions and hydroxyl, peroxyl, alkoxyl, and nitric oxide (•NO) radicals, through a mechanism that involves the transfer of a hydrogen (H) atom to the radical stabilizing it [51]. Furthermore, they may also act as chelators of transition metal ions, thus, preventing the iron- and copper-catalyzed formation of initiating radical species [52]. Structural criteria for optimal scavenging activity in the case of flavonoids are the presence of a catechol group in the B-ring, a 2,3-double bond conjugated with a 4-oxo function in the C-ring, and a 3- (and 5-) hydroxy group, as they provide extensive electron delocalization over the three-ring system and confer higher stability to the produced radical [53]. It is generally assumed that flavonoids that lack some of these features possess weaker antioxidant activity. As for proanthocyanidins, the degree of polymerization also appears to have an influence on the radical scavenging properties. In this case, extensive conjugation between the OH at C-3 and catechol groups of the B-ring together with abundant 4-8 linkages confer these oligo/polymers with enhanced radical scavenging ability [54]. Nevertheless, although they may behave as antioxidants in vitro, most polyphenols are rarely bioavailable and are largely biotransformed in the organism. Except for a limited number of compounds, intact polyphenols as they occur in food are not found in blood but are instead found in the form of phase II or colonic metabolites, which are the ones able to reach the biological targets. These metabolites are chemically and, in many instances, functionally distinct from the dietary form, and such features highlight their bioactivity [55].

Maximum levels of phenolic metabolites in plasma are reached 1–2 h after consumption in the case of conjugated metabolites derived from the absorption in the small intestine, and between 6 and 24 h for metabolites produced by the gut microbiota. Even at the highest levels reported in plasma (low micromolar values), the concentrations are far below those of other antioxidants, such as urate, α-tocopherol, or ascorbate, which are present in blood and/or in the intracellular milieu in micromolar and even millimolar ranges [56]. Furthermore, the existence of substituents in the hydroxyl groups (e.g., glycosylation, sulphation, or methylation) generally decreases the antioxidant capacity in relation to the parent polyphenols, so that conjugated metabolites possess significantly lower capacity for donating hydrogen ions and scavenging free radicals compared to that of the parent compounds [57,58]. Nevertheless, it is necessary to take into account that in vivo situations a mixture of metabolites would exist and that the behavior of such a combination might differ from that of the individual compounds. In a recent study on the conjugated metabolites of quercetin detected in pig urine following intake of quercetin-rich onion dry skin, Wiczkowski et al. [59] found that, whereas, quercetin aglycone possessed higher antioxidant activity than each of the individual conjugated metabolites, the pool of metabolites showed higher antioxidant capacity than that of the individual metabolites or quercetin, isorhamnetin, and their glycosides. On the other hand, different cells, including liver, blood, and kidney cells, contain enzymes capable of deconjugating the sulphated and glucuronidated forms of polyphenols [60]. Deconjugation can also be produced in inflammation situations, as neutrophils and macrophages express β-glucuronidase activity [61]. Thus, in some circumstances, flavonoid aglycones and/or their methylated derivatives could be released in some target sites and may constitute the actual in vivo active forms [62]. In studies on both isolated rat mesenteric bed and spontaneously hypertensive rats, it was shown that quercetin-3-*O*-glucuronide, the main conjugated metabolite of quercetin, was able to produce a significant decrease in blood pressure following deconjugation to the aglycone at the vascular wall level [63,64]. This anti-hypertensive response might be related, at least in part, to an inhibitory effect of quercetin in the -adrenergic-induced hypercontractile response in resistance arteries [63,64].

In spite of the challenges posed by their limited bioavailability, mechanisms based on antioxidant activity have still been considered to explain some of the in vivo effects of flavonoids. Thus, Laranjinha and co-workers [65,66] explored the hypothesis that circulating polyphenols might overcome the isotropic dilution in blood plasma by binding to biomembranes and lipoproteins. By accumulating at lipid:water interfaces, they could achieve local concentrations high enough to afford a confined antioxidant protection. Whereas, the interaction with lipids seems to be mostly dependent on the hydrophobic characteristics of the molecule, and in the case of proteins, the availability of phenolic hydroxyl groups that allow hydrogen bonding may also be important. Proline-rich proteins, such as saliva proteins, seem preferential targets for these interactions. On the other hand, a significant number of enzyme activities have been reported to be inhibited by polyphenols. Of particular interest, because of their role in inflammatory conditions, is the reductive inactivation of lipoxygenases, cyclo-oxygenases, myeloperoxidase, or xanthine oxidase, which, in view of their pro-oxidant activity, may be regarded as an indirect antioxidative action [67].

In recent years, increasing attention has also been paid to the possibility of phenolic compounds acting as potential modulators of intracellular signaling cascades vital to cellular function beyond their classical antioxidant capacity. Thus, the biological effects of flavonoids could be mediated by their ability to modulate the activity of both protein and lipid kinase signaling cascades (e.g., mitogen-activated protein (MAP) kinases, Akt/PKB) and transcription factors (e.g., Nrf2, AP-1, NF-κB) that are crucial players in inflammatory and immune responses [68]. Also, dietary phytochemicals, including flavonoids, have been shown to be able to modulate the Keap1/Nrf2 system that plays a key role in cellular protection by regulating many antioxidant and detoxifying enzyme genes through the antioxidant response element (ARE) [68].

The modulation of nitric oxide (•NO) metabolism is another possible mechanism that could contribute to the explanation of the in vivo activity of polyphenols. In the human organism, NO is produced not only through enzymatic pathways (eNOS, iNOS) but also by the reduction of dietary nitrate and nitrite in the stomach. After a meal, high concentrations of both nitrite and polyphenols may occur at this location. Taking into account their reductive ability, nitrite reduction to •NO is likely to occur at the acidic gastric pH, which would constitute a large source of this molecule independent of its enzymatic synthesis from nitric oxide synthase [51]. What is more, this effect would be produced by the compounds in the form in which they are conveyed in food. Indeed, the ability of wine polyphenols to promote the production of NO from nitrite was shown both in vitro and in human volunteers by measuring NO in the air expelled from the stomach [69]. In the stomach, dietary polyphenols may not only promote nitrite reduction to NO but also embark on a complex network of chemical reactions to produce nitrogen oxides with signaling functions. Thus, local and systemic effects of NO could be, in this sense, triggered by dietary flavonoids. Thus, the biochemistry of polyphenols in the stomach and intestine, in connection with the nitrate-nitrite-NO pathway, could constitute a shortcut for the biological effects of these molecules with an impact on human health [70].

Overall, what seems clear is that the notion of polyphenols acting as mere antioxidants is unlikely to be the sole explanation for their putative health effects. Modulation of redox signaling, entailing the modification of gene expression and of enzymatic activity, as well as interference by nitric oxide metabolism are mechanisms that might be involved in the biological effects of polyphenols, contributing to explain their influence on health, beyond direct antioxidant activity [51].

## 4. Effects of Wine Polyphenols at the Local Level

### 4.1. Microbiota Modulation by Wine Polyphenols

It is accepted today by the scientific community that the homeostasis and resilience of the intestinal microbiota is associated with the higher microbial diversity characteristic of young and healthy individuals, whereas inflammatory and metabolic disorders coincide with changes in the composition and/or functions of the intestinal microbiota [71].

Bacteria dominate the gut microbiota, being represented principally by the phyla Firmicutes and Bacteroidetes, and by secondary phyla such as Actinobacteria, Proteobacteria, Synergistetes, Fusobacteria, and Verrucomicrobia [14]. Among the main representative genera of these phyla, *Bacteroides* sp., *Faecalibacterium* sp., *Blautia* sp., *Prevotella* sp., *Clostridium* sp., *Ruminococcus* sp., and *Bifidobacterium* sp. are noteworthy due to their relatively high abundance, and indeed each and every one of us harbors several grams of one or more of these bacterial genera. In turn, it is assumed that several hundred species-level bacteria assemble in each individual in highly variable proportions, resulting in an individual microbial composition that remains stable over time [72]. The temporal stability of the intestinal ecosystem is probably maintained by host-encoded mechanisms in parallel with colonization resistance, as a balanced climax community is not susceptible to new (invading) species. Quite recently, a new classification has been proposed, according to which all the inter-individual variability of the intestinal microbiota can be classified into three groups, the so-called “enterotypes”, which are defined as a network of co-abundant microbial populations dominated by the prominent presence of one of these three genera: *Ruminococcus*, *Bacteroides,* and *Prevotella* [73]. This classification has been a source of controversy, since some authors consider “enterotypes” a very simplistic model, since the whole intestinal microbiota complexity cannot be reduced into three groups [74].

Although numerous in vitro studies suggest that wine polyphenols can affect colonization and the composition of the intestinal microbiota, only a few human studies have investigated the modulating effect on intestinal microbiota derived from moderate wine consumption. In a randomized, crossover, and controlled trial, the effects of the intake of red wine, de-alcoholized red wine and gin were compared [75,76]. After the red wine period (272 mL/day, 20 days, 8 volunteers), an increase was observed in: populations of Proteobacteria, Fusobacteria, Firmicutes, and Bacteroidetes, at phylum level; *Enterococcus*, *Prevotella*, *Bacteroides* and *Bifidobacterium*, at genera level; and the *Blautia coccoides*-*Eubacterium rectale* group and *B. uniformis* and *Eggerthella* species group. The impact of moderate regular consumption of red wine on the fecal microbial metagenomic profile of healthy individuals has also been investigated using 16S rRNA gene sequencing of fecal samples [77]. An increase in microbial diversity after wine intake was observed. Some differences in minority microbial groups related to phenolic metabolism phenotypes (“metabotypes”) were found, but inter-individual variability was the strongest and distinguishing feature. On the other hand, the consumption of wine, determined by a food frequency questionnaire (FFQ), showed an association with high polyphenol content and microbial abundance and/or diversity [78,79]. In particular, an increase in the abundance of *Faecalibacterium prausnitzii*, which has anti-inflammatory properties, was observed [79].

To date, studies about how diet influences intestinal microbiota have been mainly focused on determining changes in microbiota composition. However, a new perspective based on functional adaptation of intestinal microbiota to diet changes is emerging [80]. In fact, the Human Microbiome Project (HMP), whose primary goal was to establish the taxonomic entities and composition that constitute a healthy gut microbiota in humans, is currently focused on multiple functional approaches, including transcriptomics (RNA-seq), metabolomics, and proteomics [81]. This new trend toward functional measurements in the studies on gut microbiota mirrors the current emphasis on integrating host-level functional events by using animal models and human studies. A clear example is the study conducted by David et al. [82], in which the effects of an exclusively plant or animal diet (5 days) on the composition and metabolic activity of the microbiota were compared. The results showed that the microbial gene expression profile clustered according to diet rather than individuals, thus, demonstrating a fast functional adaption of intestinal microbiota to dietary changes. Recently, Wu et al. [83] compared the metabolome of healthy human vegans and omnivores sampled in an urban environment. The authors found that although there were no major differences between the two groups in terms of microbiota composition, significant changes in the profile of urinary metabolites, especially those derived from microbial metabolism, existed. In this same context, a recent study has gone one step further suggesting that changes in the diversity and function of the intestinal microbiota could be transferred to subsequent generations [84]. Using humanized mice, that study demonstrated that prolonged reduction in fiber intake leads to a progressive loss of diversity in the gut, which is increased over the time and is not recoverable after the reintroduction of dietary fiber. In turn, a loss of enzymatic activities associated with fiber degradation was observed. Based on those results, the authors suggested that restoring the intestinal microbiota to its original state through diet should be done for long periods of time, and even suggested the administration of missing bacterial groups by using probiotics.

Even being aware of the impact of diet on metabolic functions of the intestinal microbiota, it is important to note that, despite the large inter-individual variability in terms of bacterial taxonomy, the functional genetic profile expressed by the bacterial community is quite stable and similar in healthy individuals, thereby ensuring those essential functions for the survival of the host [85,86]. Therefore, the microorganisms present in smaller quantities, but developing specific functions, could be the key to understanding the individual response to consumption of bioactive compounds (i.e., polyphenols). An example of this approach is the case of urolithins, which, as previously indicated, are produced by the human gut microbiota from ellagitannins and ellagic acid. These metabolites are much better absorbed than their precursors and have been suggested to be responsible for the health effects associated with those polyphenols. The inter-individual variability observed for the production of urolithins (“metabotypes”) and their relationship with health status and dysbiosis have been noticed [49]. In the case of wine, a recent study has assessed the robustness of clinical and metabolic phenotyping, through the identification of a differential response to red wine polyphenol intake, in the improvement of cardiometabolic risk conditions. The results revealed the existence of gut microbiota-responsive phenotypes to wine polyphenol intervention, which highlights a novel metabolomic strategy for characterizing inter-individual response to dietary intervention and identification of health benefits [87]. Therefore, population stratifications according to their capacity to metabolize polyphenols seems not only to correlate with the concentration and/or functionality of the gut microbiota involved in the production of these metabolites, but also to disease biomarkers.

### 4.2. Interactions with Host Cells

Some authors have suggested that wine polyphenols could have an effect on the immune and/or inflammatory bowel response, as several studies with cellular models have revealed [88]. Moreover, intervention studies with wine in patients with inflammatory bowel disease have also been conducted, suggesting a positive effect, although the biochemical support behind that activity is limited in many cases. Swanon et al. [89] carried out a study of wine consumption (1–3 glasses/day, 7 days) with patients diagnosed with ulcerative colitis (*n* = 8) and Crohn’s disease (*n* = 6), finding a significant increase in intestinal permeability and also a significant decrease in fecal calprotectin content. As research progresses, the role of intestinal microbiota in inflammatory bowel pathologies’ development becomes more evident [90]. In this context, a human intervention study of moderate and regular red wine intake showed a significant decrease in the fecal concentration of cytokines in a subset of volunteers (*n* = 6) who had high values of these markers at the start of the intervention, though without apparent symptoms of inflammatory bowel disease [91]. The decrease was particularly remarkable for pro-inflammatory cytokines (TNF-α, IL-6, IL-8 and IFN-γ), which have been associated with mild states of intestinal inflammation [91]. What is more, a negative correlation between IFN-γ cytokines, IL-8 and IL-6 and total fecal content of phenolic metabolites for this subpopulation was found, suggesting that microbial metabolites derived from wine polyphenols may have beneficial effects in early (and still asymptomatic) stages of inflammatory bowel disease, either by a direct anti-inflammatory effect or by interaction with the intestinal microbiota action.

## 5. Future Perspectives

As discussed above, the actual mechanisms behind the systemic in vivo activity of polyphenols go beyond their antioxidant activity and are still under discussion. Interaction with signaling cascades involving cytokines and transcription factors, such as the Keap1/Nrf2-ARE system, as well as interference by nitric oxide metabolism are mechanisms that might contribute to explaining their involvement in biological effects. However, most of those processes have only been studied in vitro or in model systems and for a limited number of molecules. More knowledge has, therefore, to be accumulated concerning the bioavailability and metabolism of many polyphenols, their biological targets, and actual active forms, as well as about in vivo concentrations of the distinct phenolic metabolites and whether they could be sufficient to exert activity at the molecular level. Further intervention studies involving a range of doses and combinations and well characterized participants are thus required, targeting the different pathways or elements and with enough power to detect differences between compounds [68].

A current key issue regarding the health implications of polyphenols is their interaction with the microbiota, which has become a hot topic in order to improve nutritional strategies with potentially important health implications. Recently, the use of polyphenols together with probiotics and prebiotics (the so-called three “P” for gut health) has been proposed as a new strategy for modulating the composition and metabolic activity of gut microbiota [18]. Wine polyphenols comprising several classes of phenolic structures might be a good exponent of this potentiality.

To date, and despite the advances in the knowledge of the identification of phenolic metabolites, the specific bacterial species able to metabolize wine polyphenols in the gastrointestinal tract and the anaerobic degradation pathways remain largely unknown (see Cueva et al. [3] for review). Also, there are only a few studies screening bacteria from other sources that are also able to metabolize wine polyphenols [92,93]. Further investigations into the isolation, characterization, and production of bacteria responsible/capable of metabolizing wine polyphenols is, indeed, an area of future research.

The combination of polyphenols and probiotic bacteria is shaping up as another nutritional strategy. In fact, consumption of specific probiotic strains might improve the metabolism and bioavailability of wine polyphenols and, in turn, wine polyphenols might improve the growth and beneficial properties of probiotics in relation to intestinal health. Although evidence on this incipient topic is scarce, several commercial probiotic preparations seem to promote the metabolism of wine phenolic extracts in vitro, as recently reported [94]. Furthermore, wine phenolic compounds have shown a certain stimulatory effect on bacterial growth, and act synergistically with probiotics to inhibit the adherence of intestinal pathogens to intestinal cells, suggesting that benefits of wine polyphenols and probiotics may be enhanced by their concomitant interaction at the intestinal level [94].

Considering that the microbial metabolites are at least, in part, responsible for the health effects of polyphenols, differences in response to polyphenols intervention will be observed depending on the composition and “metabolic status” of the gut microbiota [49]. Although, in practice, the physiology and metabolic capabilities of the majority of the “healthy” microbial representatives of our gut have not yet been studied, and at the moment the scientific community has not reached a consensus on how to define our beneficial intestinal microbial fingerprinting, attempts have been made to propose new classes of specific intestinal microbiota that reflect a “healthy catabolic function” [49]. For example, the Firmicutes/Bacteroidetes ratio seems to have some health implications, in particular, higher values of this fraction have been associated with obesity and type 2 diabetes [95]. Therefore, changes in microbial communities by dietary-polyphenols, and in particular by wine polyphenols, could protect against these diseases. Also, in the case of wine consumption, there is increasing interest in *Faecalibacterium prausnitziii*, which is associated with healthy intestinal conditions in humans [79]. Therefore, the question that hangs in the air is whether the beneficial effects of wine polyphenols and derived-metabolites can be associated to a specific bacteria/microbial consortia/population.

In conclusion, scientific evidence over the last 15 years supports the important role that polyphenols exert through their interactions with gut microbiota, which impacts on intestinal health. Indeed, recent literature indicates that variations in host genetics and gut microbiota among individuals are a crucial factor in explaining polyphenol health activities. Nowadays, more and more findings are emerging that help the scientific community to better understand these targets. Categorizing the intestinal microbiota into groups that reflect both gut microbiota composition and catabolic function could be extremely helpful in personalizing/establishing polyphenol dietary strategies for the prevention and treatment of diseases associated with microbial dysbiosis, and, particularly, inflammatory bowel diseases, obesity, and metabolic syndrome. This will require a better definition of the large inter-individual variations and, most importantly, getting stratification of individuals based on specific gut microbiota functional features for obtaining positive polyphenol-mediated health effects. In this regard, even though this should be appropriately confirmed, wine and grapes, as largely available sources of polyphenols, could represent a promising strategy.

## Figures and Tables

**Table 1:** Main class of grape and wine polyphenols and their microbial-derived metabolites.

Precursors		Main Metabolites Identified
Flavan-3-ols	(+)-Catechin 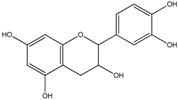	Epicatechin (in the case of procyanidin B2) 1-(3′,4′-Dihydroxyphenyl)-3-(2″,4″,6″-trihydroxyphenyl)propan-2-ol 1-(3′-Hydroxyphenyl)-3-(2″,4″,6″-trihydroxyphenyl)-propan-2-ol 5-(3′4′-Dihydroxyphenyl)-γ-valerolactone 5-(3′,4′-Dihydroxyphenyl)-valeric acid 4-Hydroxy-5-(3′4′-dihydroxyphenyl)-valeric acid 3,4-Dihydroxyphenylpropionic acid 3-Hydroxyphenylpropionic acid 3,4-Dihydroxybenzoic acid 3-Hydroxybenzoic acid
	(+)-Epicatechin 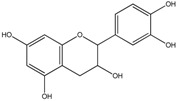	
	Procyanidin B2 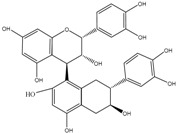	
Anthocyanins	Malvidin-3-*O*-glucoside 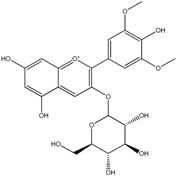	Syringic acid Methyl gallic acid Gallic acid 2,4,6-Trihydroxybenzaldehyde Phloroglucinol
	Peonidin-*O*-glucoside 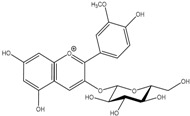	Vanillic acid Protocatechuic acid 2,4,6-Trihydroxybenzaldehyde Phloroglucinol
Flavonols	Quercetin-3-glucoside 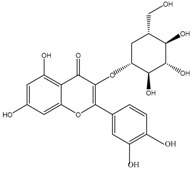	3-(3,4-Dihydroxyphenyl)propionic acid 2-(3,4-Dihydroxy)-phenylacetic acid Protocatechuic acid
	Kaempferol 3-glucoside 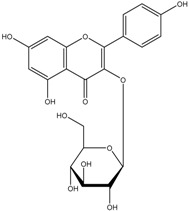	2-(4-Hydroxyphenyl)propionic acid 2-(3,4-Dihydroxy)-phenylacetic acid Protocatechuic acid
